# Supplementary material for: Putative Biomarkers for Prognosis, Epithelial-to-Mesenchymal Transition, and Drug Response in Cell Lines Representing Oral Squamous Cell Carcinoma Progression
Source: Genes (Basel). 2025 Feb 9;16(2):209. doi: 10.3390/genes16020209 (PMC11855662; doi:10.3390/genes16020209)
Supplement: Supplementary file 1 [file genes-16-00209-s001.zip › Genes Supplementary Revised.pdf]

SUPPLEMENTARY MATERIAL FOR

Putative Biomarkers for Prognosis, Epithelial to Mesenchymal Transition, and Drug Response in Cell Lines Representing Oral Squamous Cell Carcinoma Progression

Mohamad Z. Hamoui, Shuaa Rizvi, Hilal Arnouk, and Cai M. Roberts

Supplementary Tables

Cell Line	Origin	Source	Growth Medium
PGK	Normal tissue from 60 y.o. female	ATCC	Dermal Cell Basal Medium, Keratinocyte Growth Kit (ATCC)
DOK	Dysplastic tissue from tongue of 57 y.o. male	ECACC	DMEM + 10% FBS + 5µg/mL hydrocortisone + 1% Penicillin/Streptomycin
SCC-25	Squamous cell carcinoma from tongue of 70 y.o. male	ATCC	DMEM:F12 + 10% FBS + 400ng/mL hydrocortisone + 1% Penicillin/Streptomycin
Detroit 562	Pleural effusion from female with metastatic oropharyngeal carcinoma	ATCC	EMEM + 10% FBS + 1% Penicillin/Streptomycin

Table S1. Cell line information.

Protein	Name / Clone	Supplier	Catalog No.	Dilution
Stratifin	14-3-3 sigma	Invitrogen	MA5-11663	1:1000
GSTP1	3F2	Cell Signaling	3369S	1:2000
TPM1	--	Proteintech	28577-1-AP	1:4000
E-Cadherin	24E10	Cell Signaling	3195T	1:1000
N-Cadherin	13A9	Novus	NBP1-48309SS	1:1000
Fibronectin	EP5	Santa Cruz Biotechnology	sc-8422	1:250
β-Actin	--	Santa Cruz Biotechnology	sc-47778	1:500

Table S2. Primary antibody information.

Supplementary Figure S1

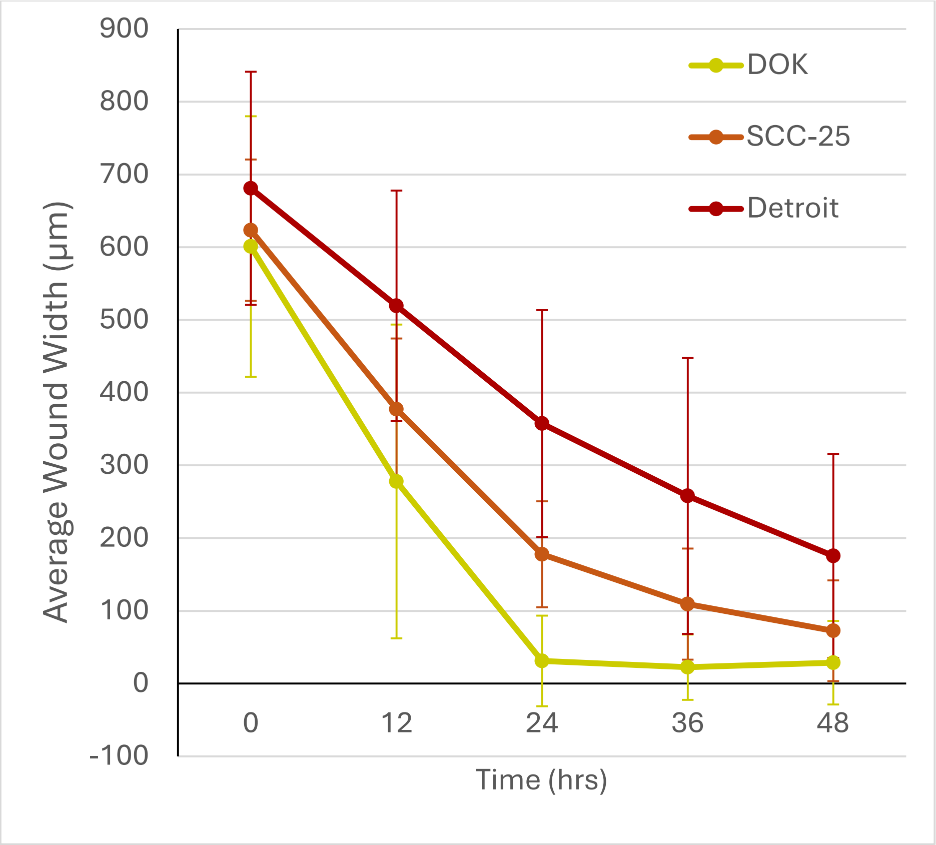

Supplementary Figure S1: Quantitation of wound healing assays. One representative well from each of four independent experiments per cell line was analyzed. The wound gap was measured every 12 hours at three points in each image (left, center, right) and these were averaged to give an average width. Graph represents mean and standard deviation of these average widths across four independent experiments.

Supplementary Figure S2

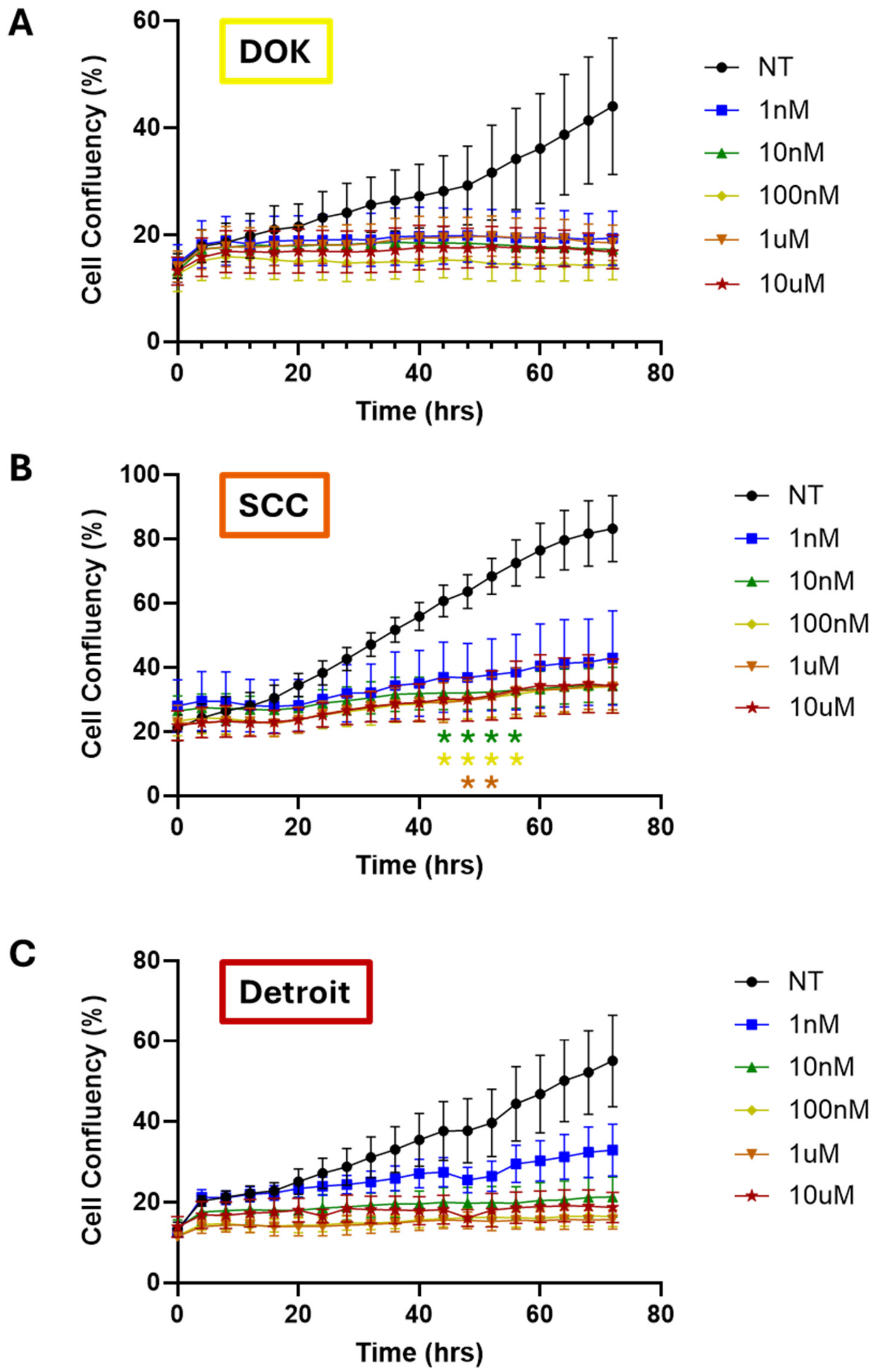

Supplementary Figure S2: Full time course data for Celloger cell proliferation assay. Cell confluence at four-hour intervals is shown for the indicated doses of docetaxel in **A.** DOK, **B.** SCC-25, and **C.** Detroit 562 cells. In **B.**, * p < .05 for the dose indicated by the color of the *.

Legends for Supplementary Movies

Supplementary Movies S1-S3: 24 hour time lapses of **S1.** DOK cells, **S2.** SCC-25 cells, and **S3.** Detroit 562 cells in wound healing assay. Each frame represents one hour of time. Time stamps appear in the top left and scale bar (200um) appears in the lower left. Each line largely fills the wound by 24 hours, but Detroit cells exhibit pronounced projections at the leading edge that are absent in the other two lines.

Supplementary Movies S4-S6: 72 hour time lapses of **S4.** DOK cells, **S5.** SCC-25 cells, and **S6.** Detroit 562 cells in drug response assays. Each frame represents four hours elapsed. Time stamps appear in the top left and scale bar (200um) appears in the lower left. Sytox green dye marks cell death. Fewer green cells relative to the total number are seen in Detroit 562, suggesting inhibition of cell division rather than widespread cell death in this line.
